# My anomaly

**DOI:** 10.1017/S1478951526102521

**Published:** 2026-04-28

**Authors:** Ijeoma Julie Eche-Ugwu

**Affiliations:** 1 Phyllis F. Cantor Center for Research in Nursing and Patient Care Services, Dana-Farber Cancer Institute, USA; 2 Harvard Medical School

## My anomaly

I often wonder,
is it my hair?
the bends of the braids
that upends the curled grades
and weaves my back
àzụm
my native tongue

I often wonder
is it my stance?
the calf that affirms
and seeks to assign
the ground beyond frown
over raffia palm, I float
in and out of time

I often wonder
is it my laughter?
the crackling troughs
that uncover the blows
the pause,
the shaded cracks
and joy claps

I often wonder
is it my skin?
coal scorched within
thinning in fold
sinning in roads
raptured by trench town yard
tunes to Marley and Tosh

I often wonder
is it my response?
that staples silenced rooms
while nursing brooms
the anomaly I see
that I resist
as I exist

